# Supplementary material for: Immunochemical characterisation of styrene maleic acid lipid particles prepared from Mycobacterium tuberculosis plasma membrane
Source: PLoS One. 2023 Jan 6;18(1):e0280074. doi: 10.1371/journal.pone.0280074 (PMC9821473; doi:10.1371/journal.pone.0280074)
Supplement: S1 Raw images — (PDF) [file pone.0280074.s001.pdf]

SUPPLEMENTARY INFORMATION

Immunochemical characterisation of styrene maleic acid lipid particles prepared from *Mycobacterium tuberculosis* plasma membrane

Sudhir Sinha, Shashikant Kumar, Komal Singh, Fareha Umam, Vinita Agrawal, Amita Aggarwal, Barbara Imperiali

S5 Raw Images. Original unprocessed images for Figures 1A, 1B, 3A, 3B, 4, 5B and 6D.

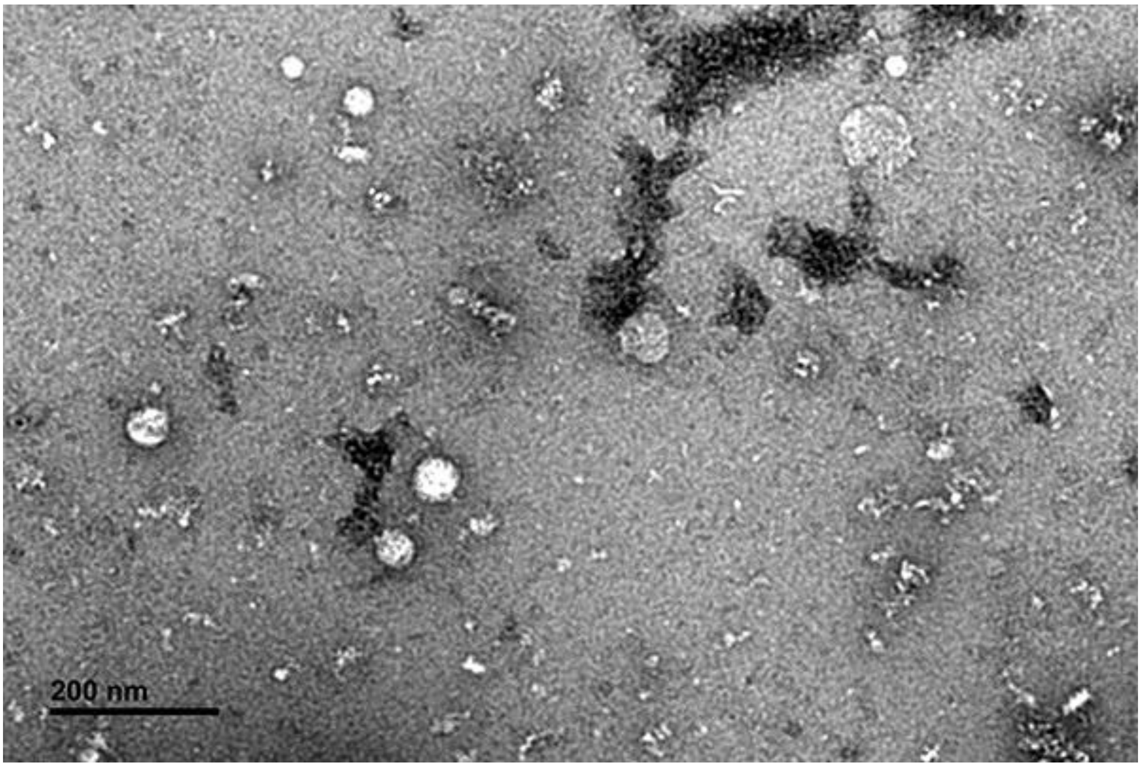

[Unprocessed Figure 1A]

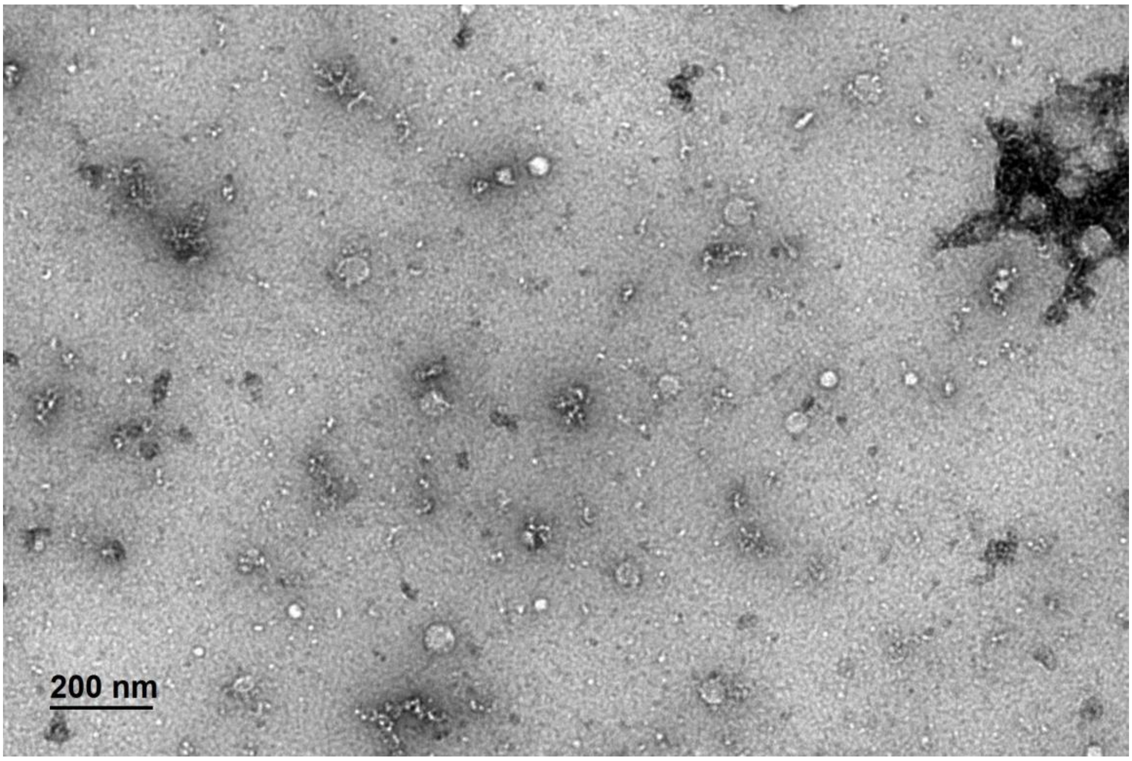

[Unprocessed Figure 1B]

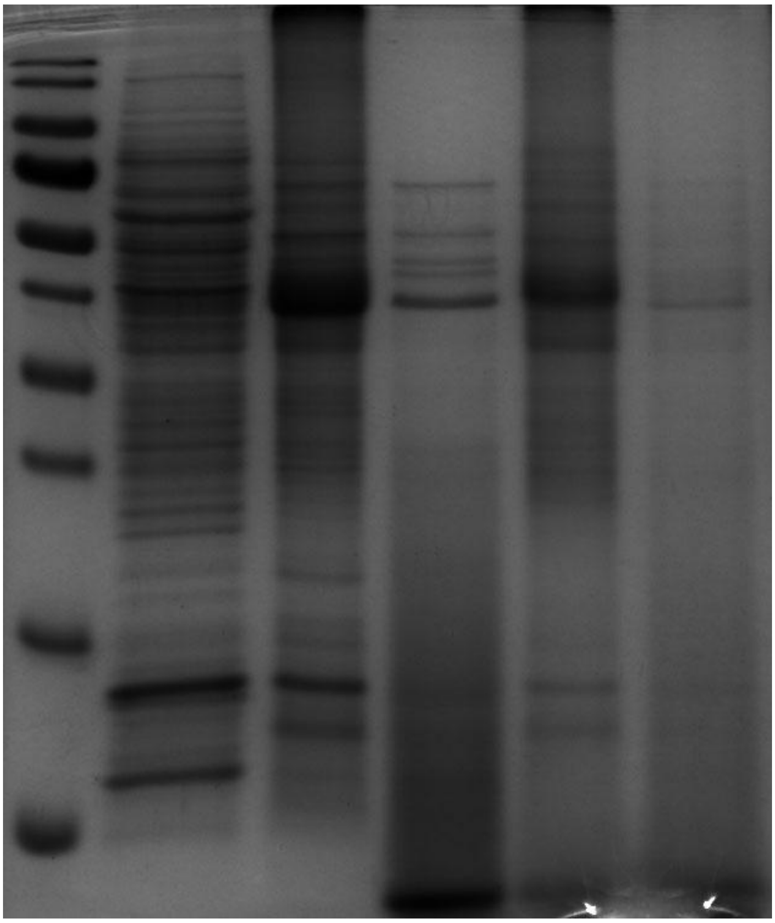

[Unprocessed Figure 3A]

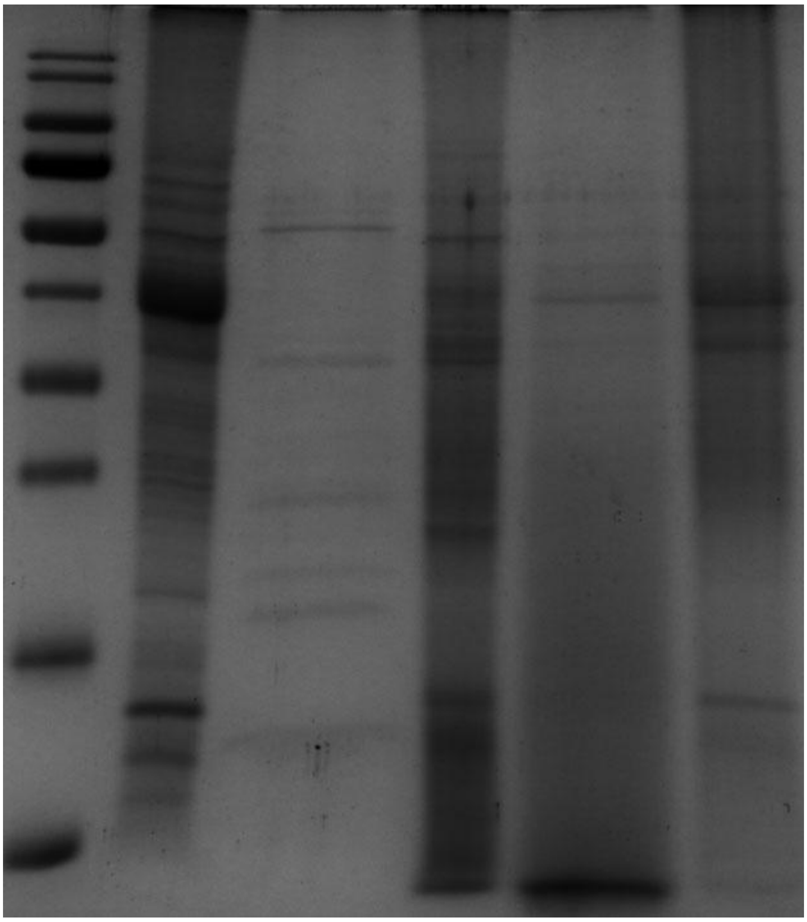

[Unprocessed Figure 3B]

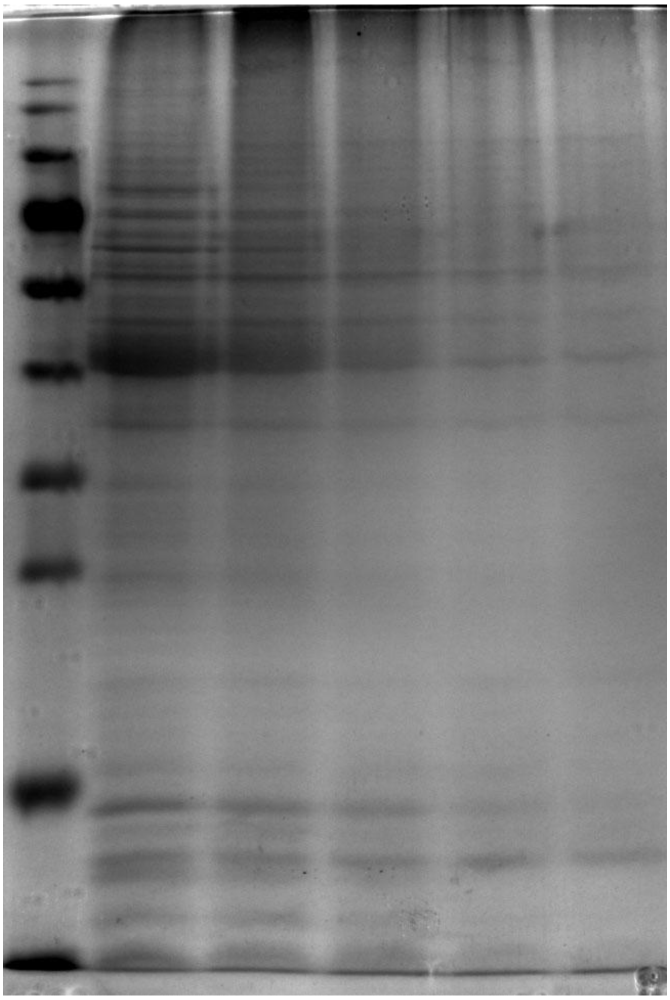

[Unprocessed Figure 4]

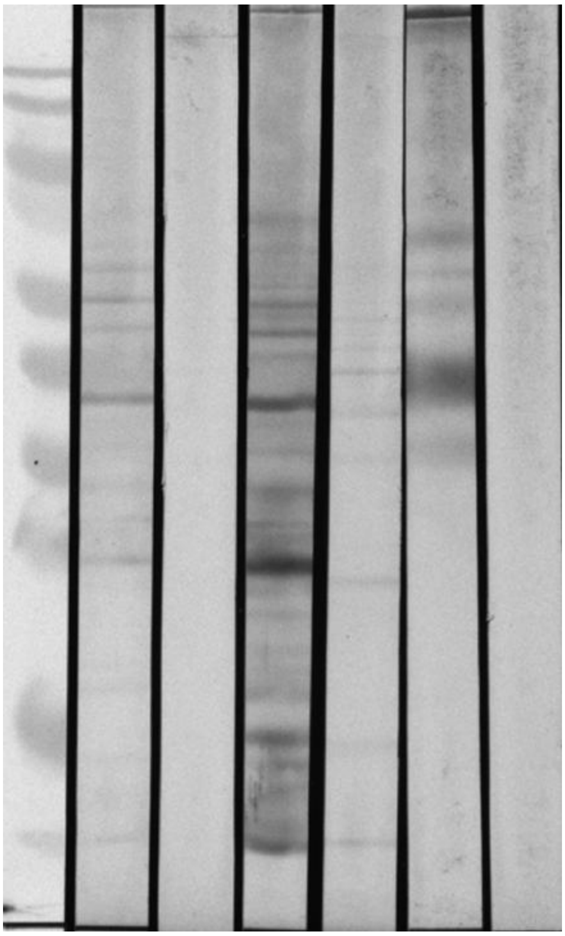

[Unprocessed Figure 5B]

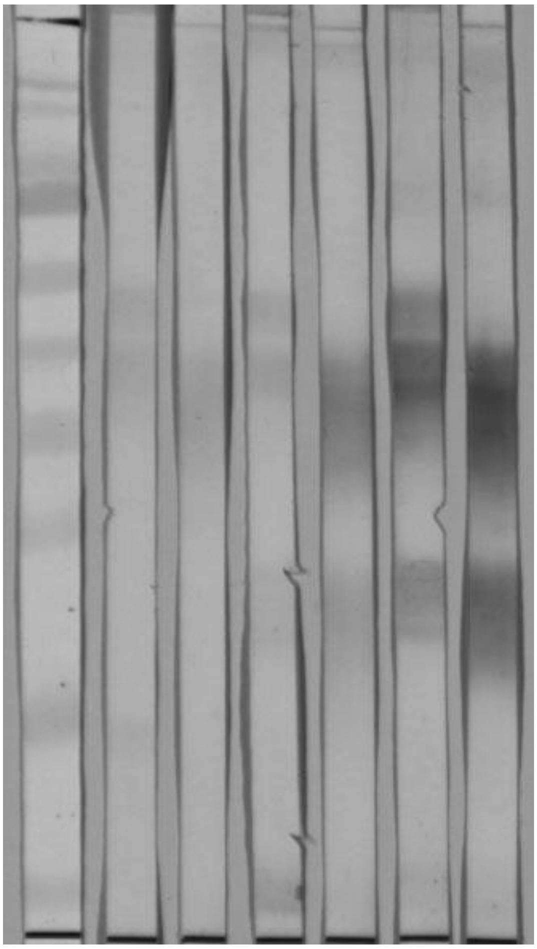

[Unprocessed Figure 6D]
