# Supplementary material for: Immunochemical characterisation of styrene maleic acid lipid particles prepared from Mycobacterium tuberculosis plasma membrane
Source: PLoS One. 2023 Jan 6;18(1):e0280074. doi: 10.1371/journal.pone.0280074 (PMC9821473; doi:10.1371/journal.pone.0280074)

SUPPLEMENTARY INFORMATION

Immunochemical characterisation of styrene maleic acid lipid particles prepared from *Mycobacterium tuberculosis* plasma membrane

Sudhir Sinha, Shashikant Kumar, Komal Singh, Fareha Umam, Vinita Agrawal, Amita Aggarwal, Barbara Imperiali

---

S1 Figure (A-F). Measurement of diameter of discoid MtM-SMALPs in TEM images.

A

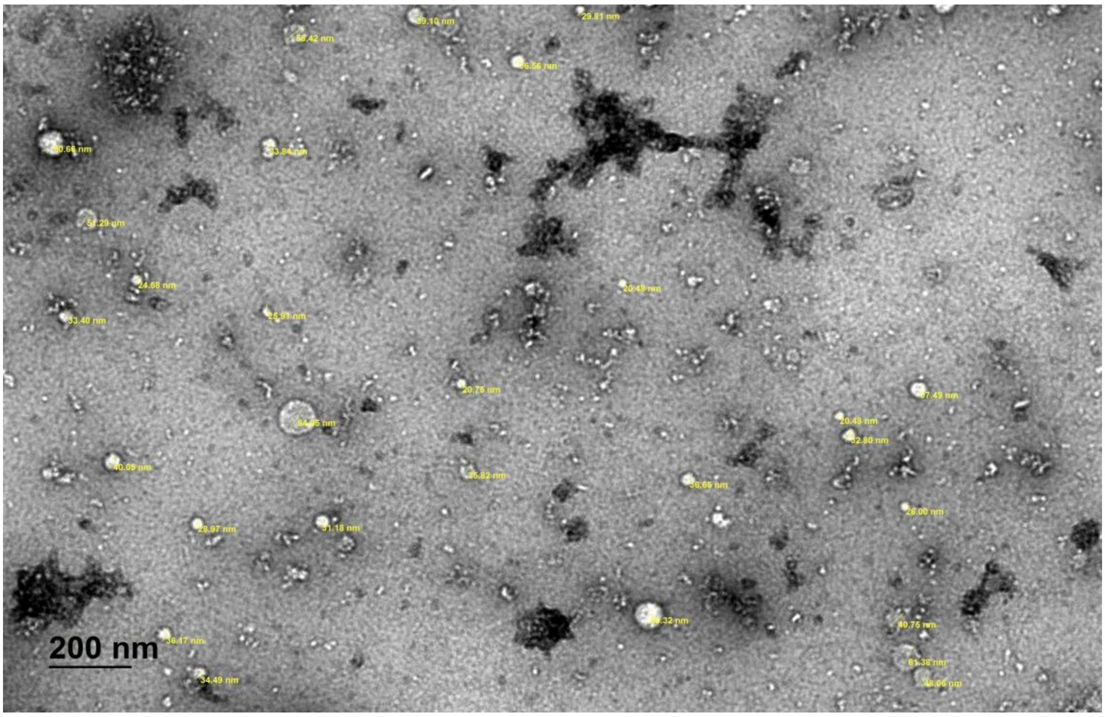

B

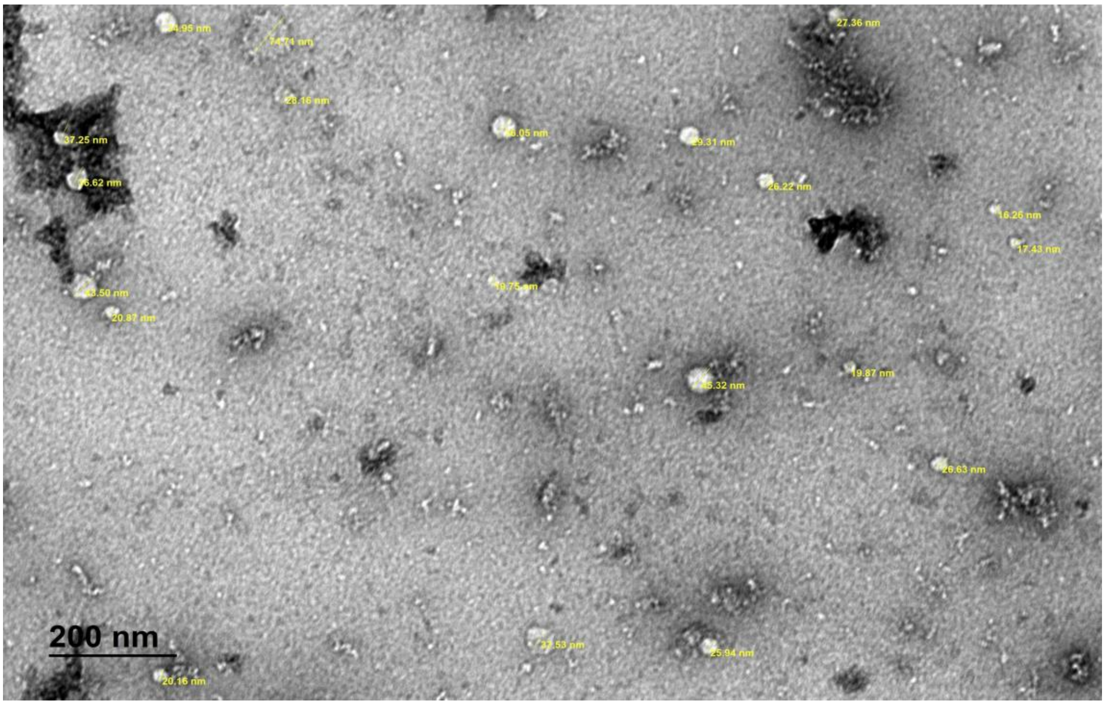

C

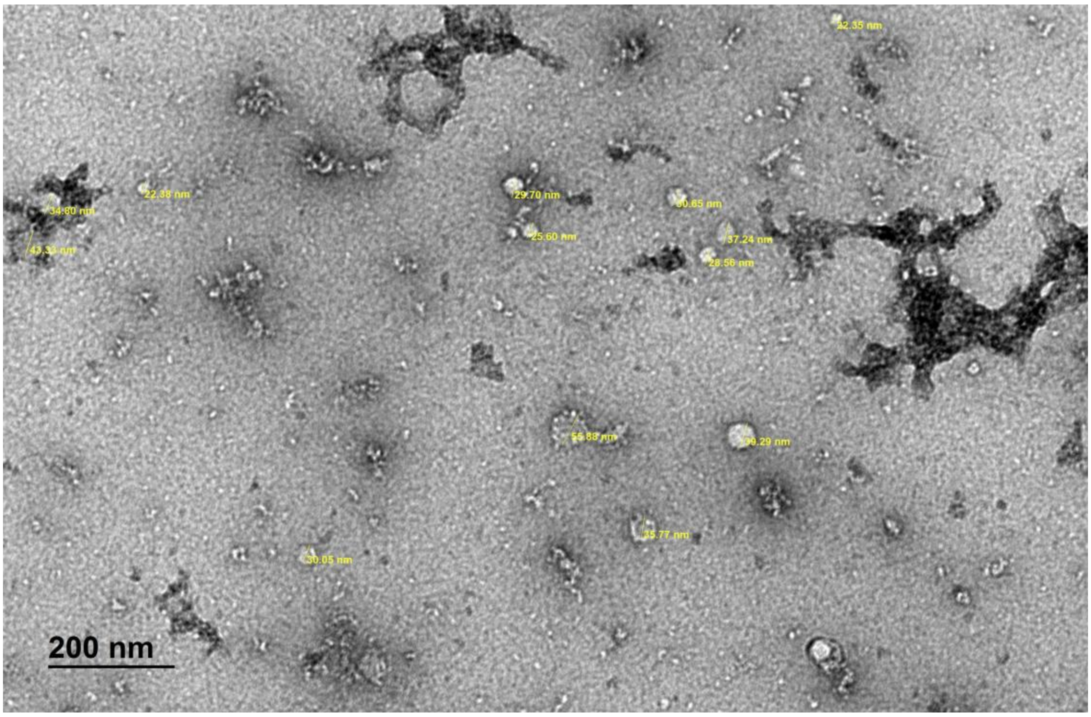

D

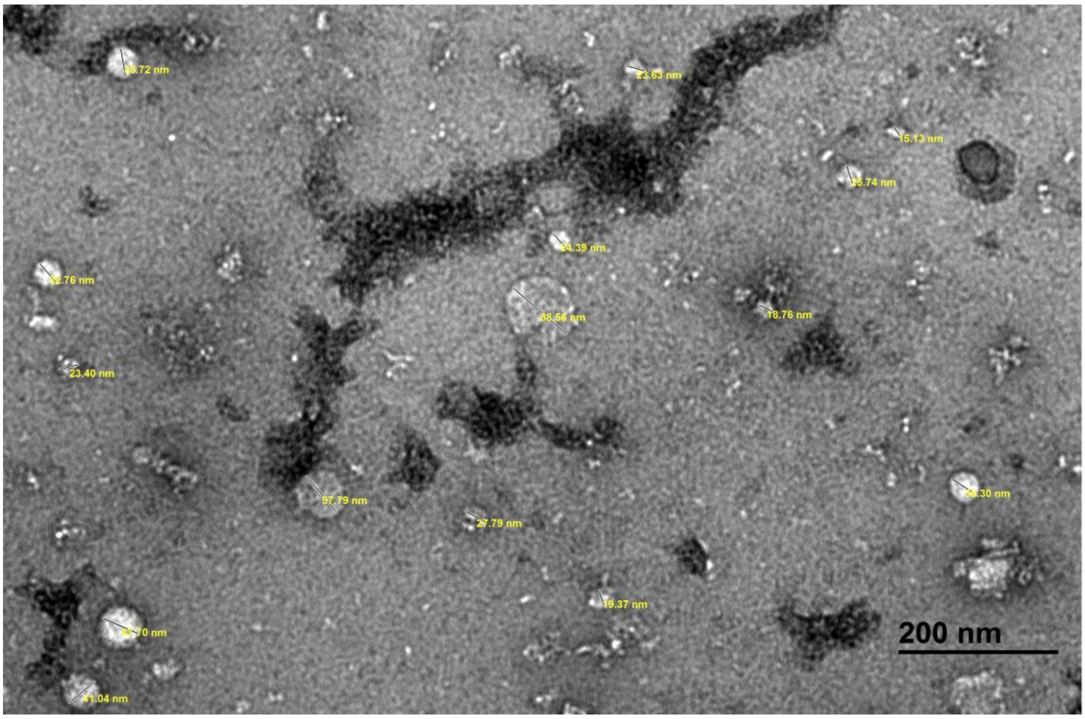

E

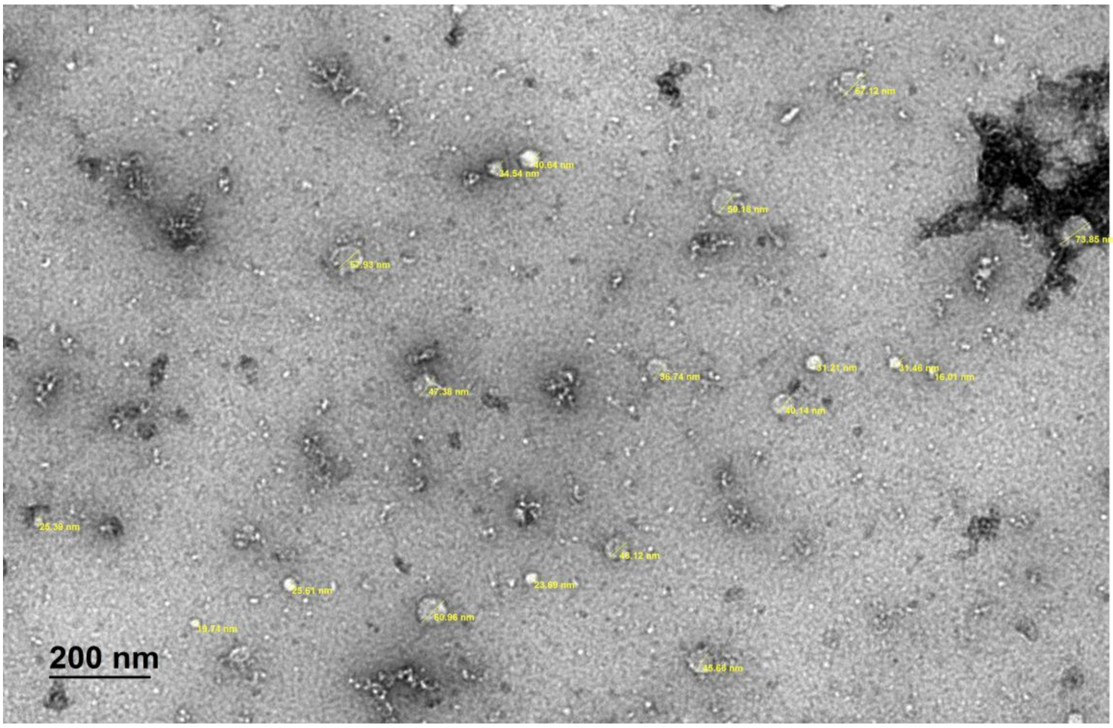

F

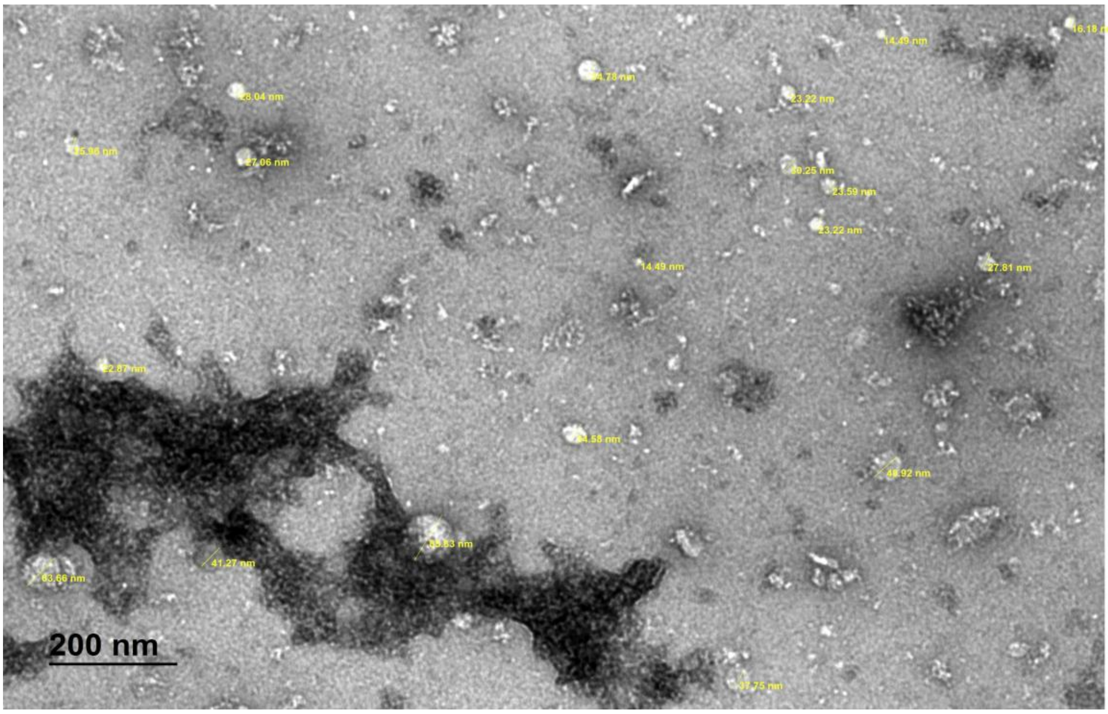

Supplement: S1 Fig — (A-F). Measurement of diameter of discoid MtM-SMALPs in TEM images. (PDF) [file pone.0280074.s002.pdf]
