# Supplementary material for: Immunochemical characterisation of styrene maleic acid lipid particles prepared from Mycobacterium tuberculosis plasma membrane
Source: PLoS One. 2023 Jan 6;18(1):e0280074. doi: 10.1371/journal.pone.0280074 (PMC9821473; doi:10.1371/journal.pone.0280074)

SUPPLEMENTARY INFORMATION

Immunochemical characterisation of styrene maleic acid lipid particles prepared from *Mycobacterium tuberculosis* plasma membrane

Sudhir Sinha, Shashikant Kumar, Komal Singh, Fareha Umam, Vinita Agrawal, Amita Aggarwal, Barbara Imperiali

S3 Figure. TEM image of MtM-SMALPs after lyophilisation and reconstitution with water.

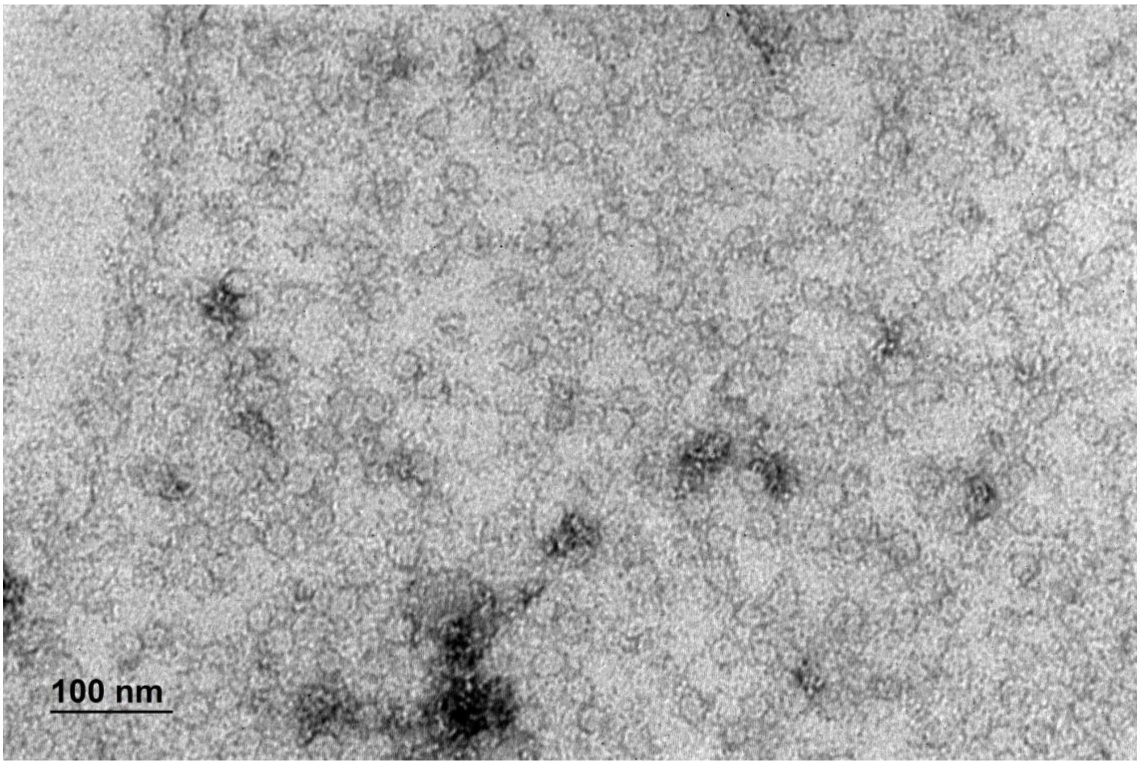

Supplement: S2 Fig — (PDF) [file pone.0280074.s003.pdf]
