# Supplementary material for: Immunochemical characterisation of styrene maleic acid lipid particles prepared from Mycobacterium tuberculosis plasma membrane
Source: PLoS One. 2023 Jan 6;18(1):e0280074. doi: 10.1371/journal.pone.0280074 (PMC9821473; doi:10.1371/journal.pone.0280074)
Supplement: S1 Table — (PDF) [file pone.0280074.s005.pdf]

SUPPLEMENTARY INFORMATION

Immunochemical characterisation of styrene maleic acid lipid particles prepared from *Mycobacterium tuberculosis* plasma membrane

Sudhir Sinha, Shashikant Kumar, Komal Singh, Fareha Umam, Vinita Agrawal, Amita Aggarwal, Barbara Imperiali

---

S2 Table. Diameters (nm) of discoid nanoparticles visualised in S1 Figure (A-F)

A	B	C	D	E	F
60.7	37.25	34.8	35.72	25.39	25.96
51.29	36.62	43.33	32.76	19.74	22.87
24.58	43.5	22.38	23.8	57.93	63.66
33.4	20.87	30.05	47.7	25.61	28.04
40.05	34.95	29.7	41.04	40.64	27.06
28.97	74.71	25.6	57.79	34.54	41.27
36.17	28.16	55.88	23.63	47.38	85.83
34.49	20.16	35.77	24.39	60.96	34.78
58.42	36.05	30.65	88.56	23.69	14.49
33.84	19.75	37.24	27.79	50.18	34.58
25.91	37.53	28.56	19.37	36.74	37.75
84.05	27.36	39.29	15.13	48.12	14.49
31.18	29.31	22.35	25.74	45.66	23.22
39.1	26.22		18.76	67.12	30.25
36.56	45.32		36.3	31.21	23.59
20.75	19.87			40.14	23.22
35.82	25.94			16.01	48.92
29.81	16.26			73.85	16.18
20.48	17.43				27.81
36.65	26.63				
69.32					
20.48					
32.8					
37.49					
26					
40.75					
61.38					
48.06					
