# Supplementary material for: Immunochemical characterisation of styrene maleic acid lipid particles prepared from Mycobacterium tuberculosis plasma membrane
Source: PLoS One. 2023 Jan 6;18(1):e0280074. doi: 10.1371/journal.pone.0280074 (PMC9821473; doi:10.1371/journal.pone.0280074)
Supplement: S2 Table — (PDF) [file pone.0280074.s006.pdf]

SUPPLEMENTARY INFORMATION

Immunochemical characterisation of styrene maleic acid lipid particles prepared from *Mycobacterium tuberculosis* plasma membrane

Sudhir Sinha, Shashikant Kumar, Komal Singh, Fareha Umam, Vinita Agrawal, Amita Aggarwal, Barbara Imperiali

---

S6 Tables. Raw data for Figures 5A, 6A, 6B and 6C

[Raw data for Figure 5A]

	MtM	SMALP
PRA	0.95	0.4
Ag85	0.64	0.13
PstS	1.09	0.38
LpqH	0.94	0.23
Acr	0.87	0.04
35kd_ag	0.26	0
LAM[a]	1.19	0.15
LAM[b]	0.74	0.24

[Raw data for Figure 6A]

Medium	SMA	MtM	SMALP
0.3	0.02	24	16.8
0.1	0.1	2.2	0.9
0.1	0.2	0.5	2.1
0.01	0.01	0.3	0.1
0.01	0.1	0.6	0.4
0.01	0.3	0.6	0.3

[Raw data for Figure 6B]

Medium (IFNg)	SMA (IFNg)	MtM (IFNg)	SMALP (IFNg)	Medium (TNFa)	SMA (TNFa)	MtM (TNGa)	SMALP (TNFa)
0.01	0.01	28600	30000	0.01	0.01	0.01	8600
0.01	0.01	3900	1500	0.01	0.01	0.01	2300
0.01	0.01	2000	4100	0.01	0.01	0.01	2800
0.01	0.01	1400	1400	0.01	0.01	0.01	0.01
0.01	0.01	800	1800	0.01	0.01	0.01	0.01
0.01	0.01	0.01	0.01	0.01	0.01	0.01	0.01

[Raw data for Figure 6C]

MtM	SMALP
0.98	0.05
0.94	0.05
0.91	0.05
0.92	0.25
0.94	0.13
1.02	0.41
